# Genome-Wide Association Studies Reveal Key Candidate Genes for Egg Weight and Quality Components in an F_2_ Japanese Quail Resource Population

**DOI:** 10.3390/vetsci13090967

**Published:** 2026-09-15

**Authors:** Natalia A. Volkova, Michael N. Romanov, Polina V. Larionova, Nadezhda Yu. German, Ludmila A. Volkova, Alexander A. Sermyagin, Alexey V. Shakhin, Darren K. Griffin, Johann Sölkner, Natalia A. Zinovieva

**Affiliations:** 1 L. K. Ernst Federal Research Center for Animal Husbandry, Dubrovitsy, Podolsk Urban Okrug 142132, Russia; natavolkova@inbox.ru (N.A.V.); volpolina@mail.ru (P.V.L.); ngerman9@gmail.com (N.Y.G.); ludavolkova@inbox.ru (L.A.V.); alexshahin@mail.ru (A.V.S.); 2 School of Natural Sciences, University of Kent, Canterbury CT2 7NJ, UK; d.k.griffin@kent.ac.uk; 3 Animal Genomics and Bioresource Research Unit (AGB Research Unit), Faculty of Science, Kasetsart University, Chatuchak, Bangkok 10900, Thailand; 4 Russian Research Institute of Farm Animal Genetics and Breeding—Branch of the L. K. Ernst Federal Research Centre for Animal Husbandry, Pushkin, St. Petersburg 196625, Russia; alex_sermyagin85@mail.ru; 5 Preimplantation Genetics Group, EGA Institute for Women’s Health, University College London, London WC1E 6HX, UK; 6 University of Natural Resources and Life Sciences Vienna, 1180 Vienna, Austria; johann.soelkner@boku.ac.at

**Keywords:** Japanese quail (*Coturnix japonica*), genotyping-by-sequencing (GBS), genome-wide association study (GWAS), single nucleotide polymorphisms (SNPs), candidate genes, egg weight, eggshell weight, albumen weight, yolk weight, albumen-to-yolk ratio

## Abstract

Identifying genetic markers for economically important egg traits is essential for advancing marker-assisted and genomic selection in poultry breeding. Here, a genome-wide association study (GWAS) was performed using genotyping-by-sequencing data to identify single nucleotide polymorphisms (SNPs) and candidate genes associated with egg weight (EW) and its constituent components. We evaluated 102 laying Japanese quails from an F_2_ resource population generated by crossing divergent Japanese and Texas White breeds. Phenotypic traits included EW, eggshell weight (ESW), albumen weight (AW), thick albumen weight (TAW), yolk weight (YW), and albumen-to-yolk ratio (AYR). We identified 16 significant SNPs and 88 candidate genes. Notably, seven prioritized candidate genes (*NTN4*, *CLDN10*, *AGAP3*, *DUSP19*, *STAB1*, *SFMBT1*, and *RNF130*) overlapped with significant SNPs associated with EW, ESW, AW, YW, and AYR. Three pleiotropic SNPs (12:309207, 12:335220, and 12:470023) were associated with multiple traits, while the *AGAP3* gene harbored two SNPs linked to AW. Our data offer insights into the polygenic regulation governing egg component development in quails. These newly detected genomic regions and candidate loci offer valuable targets for downstream replication studies aimed at enhancing avian breeding programs.

## Simple Summary

At the current stage of poultry breeding for egg production, the development and improvement of highly productive breeds and crosses increasingly depend on incorporating modern technologies involving genomics. These include using genetic markers established by integrating the whole genome data into breeding programs. The efficiency of these methods is largely determined by identifying markers that are reliable and enable targeted selection for enhanced egg production and quality traits. This study investigated DNA markers associated with the weight parameters of the egg and its primary components—albumen, yolk, and shell—in laying Japanese quails during active egg laying. We identified specific variations in the genome sequence of quails and candidate genes linked to these traits. We thereby determined the genetic variants that cause certain traits to be expressed. These findings expand our fundamental knowledge of the genetic architecture of egg production in quails. Furthermore, the identified genetic variants represent a baseline for future population-specific studies to explore their potential utility in marker-assisted and genomic selection.

## 1. Introduction

Quail farming is a rapidly growing sector of global agriculture [1,2], with its products widely available on the consumer market. This commercial expansion is driven both by the unique biological features of the Japanese quail (*Coturnix japonica*; CJA) [3,4] and the high nutritional value and quality of quail-derived commodities, namely meat and eggs [5,6,7,8]. Compared to other poultry species, quails exhibit early sexual maturity and are relatively resilient to diverse housing conditions, making them highly efficient livestock models for food production [3,4,9,10]. Egg collection can begin as early as 5–6 weeks of age, depending on the breed [11]. Furthermore, quail eggs possess excellent nutritional profiles and an extended shelf life compared to chicken eggs [12,13,14].

In egg poultry breeding, egg production rate and egg weight (EW) are primary selection traits that have been evaluated for decades using genetics [15,16,17,18]. The number of eggs produced over a given period directly correlates with commercial profitability [1]. While EW influences the market pricing of table products, this trait is not as commercially critical in quail farming as it is in the chicken egg industry. That is, in chickens, EW strictly determines weight categories and final cost. Nevertheless, EW remains a critical selection trait that determines both marketable attributes and hatching qualities [10,19,20]. Extensive poultry research has demonstrated a significant correlation between EW and body weight of the resulting offspring [21,22,23,24,25].

The weight parameters of individual egg components—namely the eggshell, yolk, and albumen—are selection traits of equal importance to total EW [26,27,28]. The yolk is a rich source of lipids, proteins, fatty acids, and essential macro- and microelements [5,9,29,30]. These, collectively, determine the flavor profile, nutritional value, and energy density of the egg, while providing vital nutrients to the developing embryo [31,32]. The albumen supplies highly digestible amino acids necessary for skeletal muscle growth [28,31,33,34], while performing a critical role in embryonic nutrient supply and antimicrobial defense during incubation [35,36,37]. The eggshell provides mechanical protection for the egg contents and the developing embryo, minimizes moisture loss, and prevents microbial contamination across all stages from collection and transport to storage and incubation [38,39,40,41]. Notably, eggshell weight (ESW) strongly correlates with shell thickness and breaking strength [42,43].

The phenotypic expression of EW and its constituent components is influenced by a complex matrix of factors, including genotype [44], nutritional regimens [45], housing conditions [46,47,48], maternal age [49,50,51,52], and egg storage conditions [53]. The genetic basis of these weight parameters has been demonstrated across various poultry species using single nucleotide polymorphism (SNP)-based genome-wide association studies (GWAS) and quantitative trait loci (QTLs) mapping, notably in chickens [54,55,56,57], geese [58,59], ducks [60,61,62], and quails [63,64,65]. The most extensive genomic mapping for egg production and quality traits has been conducted in chickens, frequently utilizing F_2_ crossbred resource populations [66,67,68,69]. For instance, the Chicken QTLdb [70,71,72,73] archives over 590 QTLs associated with the weight metrics of chicken eggs and their primary components, including EW (486 QTLs), yolk weight (YW; 53 QTLs), albumen weight (AW; 15 QTLs), and ESW (39 QTLs) [74]. In contrast, genomic mapping and the identification of genetic markers linked to EW traits in quails remain highly limited compared to chickens [75].

The objective of this study was to identify SNPs and candidate genes associated with EW and its primary components—ESW, YW, AW, and thick albumen weight (TAW)—as well as the albumen-to-yolk ratio (AYR) in quails. Using genotyping-by-sequencing (GBS) data [76], we performed a GWAS analyzing these weight parameters in an F_2_ resource population generated by crossing divergent Japanese and Texas White quail breeds with contrasting egg production characteristics. Our findings expand fundamental insights into the polygenic mechanisms governing egg composition and production traits in laying quails during active oviposition. Furthermore, the newly identified SNPs and candidate loci provide a valuable baseline for future validation to explore their potential utility in marker-assisted and genomic selection strategies aimed at optimizing quail egg performance and quality.

## 2. Materials and Methods

### 2.1. Experimental Birds

Hatching eggs of purebred quails were purchased from Genofond LLC (All-Russian Poultry Research and Technological Institute, Sergiev Posad, Moscow Oblast, Russia). Incubation, rearing, and the generation of the F_2_ resource population were conducted at the L. K. Ernst Federal Research Centre for Animal Husbandry (LKEFRCAH; Dubrovitsy, Moscow Oblast, Russia), adhering to standard poultry management, housing, and feeding practices as described elsewhere [77,78,79]. The poultry facilities were equipped for full-cycle production, spanning from chicks to mature birds. The indoor microclimate was maintained at an air temperature of 20–25 °C and a relative humidity of 55–65%. During the laying period, a 16 h photoperiod with dim light intensity was implemented.

The birds were housed in battery cages. To ensure accurate phenotypic data collection for the GWAS analysis, female quails were housed in individual cages. This single-housing design was methodologically essential to guarantee strict egg-to-hen traceability and to accurately match individual phenotypes with genotypes. All housing conditions, stocking densities, and husbandry practices strictly adhered to national zootechnical standards and welfare guidelines to minimize bird stress. All experimental protocols were reviewed and approved by the LKEFRCAH Commission on the Ethics of Animal Experiments (Protocol No. 4, dated 13 June 2024). Group housing was utilized for the F_0_ and F_1_ parental groups, as well as for F_2_ chicks and mature birds; however, individual housing was applied to laying hens during the egg collection period. The battery cage system was divided into individual sections measuring 42 × 22.5 cm (0.09 m^2^), each equipped with one nipple drinker and a feeder. Feed and water were provided *ad libitum*. Throughout all rearing stages, the quails had unrestricted access to clean running water and commercial balanced feed formulated according to their physiological stage (rearing or laying).

To establish the F_2_ resource population, two breeds with contrasting egg production characteristics and EW—Japanese and Texas White [80,81,82]—were utilized. We previously conducted a comparative analysis of these breeds based on egg production and growth rate [76]. The Japanese breed is characterized by an earlier onset of egg production and higher egg production compared to Texas White quails. Moreover, Texas White quails surpass the Japanese breed in EW and growth rate.

A series of controlled matings between the parental breeds and their hybrids was performed according to a breeding scheme previously detailed [76,83,84,85]. Initially, Japanese quails were crossed with Texas White quails to produce F_1_ interbreed hybrids. For this purpose, four groups were formed, each containing one male and five females. The first two groups contained one male of the Texas White breed and five females of the Japanese breed, while the other two groups contained one male of the Japanese breed and five females of the Texas White breed. Each group produced 25–30 F_1_ descendants. From these individuals, eight groups were formed (F1_1–F1_8; each group contained one male and three females), implementing the exclusion of close relatives for further crosses aimed at producing F_2_ individuals. From this F_2_ generation, a total of 102 females with contrasting phenotypes in EW parameters were selected for subsequent molecular genetic analyses. For this purpose, F_2_ offspring with high and low EW were selected from each of the eight F_1_ males used in the crosses (groups F1_1–F1_8). To characterize the genetic structure of this model population, the F_2_ females were stratified into eight groups (G1–G8) based on their respective F_1_ paternal lineages.

### 2.2. Phenotypic Traits and Their Statistical Analyses

The F_2_ resource population quails (*n* = 102) were phenotyped for the weight parameters of their eggs and constituent components at 4 to 5 months of age. Specifically, the evaluated traits included EW, YW, AW, TAW, ESW, and AYR. Egg collection was performed individually for each laying hen under stable, stress-free management conditions. A minimum of 20 eggs per hen was analyzed. All egg quality assessments were conducted within 24 h of oviposition. The weights of the whole egg and its primary components (yolk, thick albumen, total albumen, and eggshell) were determined using an electronic analytical balance. AYR was calculated by dividing AW by YW.

Based on the raw experimental data, the mean values of EW and its component parameters were calculated individually for each F_2_ female. The mean value for each investigated trait was computed as the arithmetic mean of all recorded eggs, with a minimum of 20 eggs analyzed per individual hen.

To evaluate the phenotypic relationships among the studied traits (EW, ESW, YW, TAW, AW, AYR), Pearson correlation analysis was performed. The correlation matrix was constructed using the stats package within the R environment (version 4.0.0; R Core Team, Vienna, Austria; [86] (R Core Team, 2018)). The significance level for all correlation analyses was set at α = 0.05.

Heritability estimates and genetic correlations for egg weight and quality traits (EW, ESW, YW, TAW, AW, AYR) were calculated using a multi-trait mixed model based on the animal model methodology (BLUP Animal Model, BLUP AM). Variance-covariance components were estimated via the Restricted Maximum Likelihood (REML) procedure. Computations were executed using the BLUPF90 family of programs [87] (Misztal et al., 2020). The multi-trait animal model equation was defined as follows:

(1) $$y_{ijk}=\mu +{PG}_{i}+{Hatch}_{j}+{animal}_{k}+e_{ijk},$$

where *y* is the phenotypic observation of the egg production trait for the *k*-th individual from the *i*-th parental family group and the *j*-th incubation hatch batch; *µ* is the overall population mean for the studied trait; *PG* is the fixed effect of the *i*-th parental family group in the resource population; *Hatch* is the fixed effect of the *j*-th incubation hatch batch; *animal* is the random additive genetic effect of the *k*-th F_2_ individual; *e* is the random residual variance.

To assess the significance and effect size of the fixed factors included in the model on the phenotypic values of egg production and quality components in the F_2_ female quails, a General Linear Model (GLM) approach was employed, implemented in STATISTICA software (version 10.0; StatSoft Inc., Tulsa, OK, USA; [88] (StatSoft Inc., 2011)).

The narrow-sense heritability (*h*^2^) was calculated using the following formula:

(2) $$h^{2}=\frac{VarA}{VarA+VarE},$$

where *h*^2^ is the heritability coefficient; *VarA* is the additive genetic variance derived from the mixed model equation for the respective trait; *VarE’* is the residual variance.

### 2.3. Sampling and DNA Extraction

Genomic DNA was isolated from feather pulp samples collected from the F_2_ female quails using a commercial Biolabmix Kit for Animal Tissue (Biolabmix LLC, Novosibirsk, Russia). DNA was extracted from freshly obtained tissue samples directly on the day of collection. Prior to DNA extraction, the tissue samples were temporarily stored at 4 °C. The concentration of the extracted DNA solutions was quantified using a Qubit 3.0 fluorometer (Thermo Fisher Scientific, Wilmington, DE, USA). The purity of the DNA preparations, reflected by the absorbance ratio at 260 and 280 nm (OD_260/280_), was evaluated using a NanoDrop-2000 spectrophotometer (Thermo Fisher Scientific, Wilmington, DE, USA). The isolated DNA samples were subsequently stored at −20 °C until further genotyping analysis.

### 2.4. Sequencing, Genotyping and SNP Quality Control

Quail genotyping was performed via GBS following the protocol developed by Elshire et al. [89] with modifications proposed by Dodds et al. [90]. For GBS library preparation, a double-digestion approach was employed using the restriction enzymes *Pst*I and *Msp*I. Negative controls (containing no DNA templates) were included in the library. DNA fragment size selection in the range of 220–340 bp (including a 148 bp span for genomic sequence and adapters) was performed using the Pippin Prep system (Sage Science, Beverly, MA, USA). A set of 102 unique adapters from Illumina (San Diego, CA, USA) and Integrated DNA Technologies (Coralville, IA, USA) was utilized; each adapter contained a 10-nucleotide barcode differing from all others by at least three nucleotides. Sequencing was conducted on the Illumina NovaSeq 6000 platform using v1.5 reagents in single-end read mode (101 nucleotides). Initial raw data quality control was performed using the DECONVQC algorithm [91] and FastQC software (version 0.10.1; [92]).

The *Coturnix japonica* 2.0 (CJA 2.0) assembly [93], retrieved from the Ensembl 104 database (released 7 May 2021), served as the reference genome. Sequence processing was conducted using cutadapt, which was applied for adapter sequence trimming and barcode-based demultiplexing [94,95]. The quality of the resulting fastq files was further verified with FastQC. Read alignment to the reference genome was performed using bowtie2 (version 2.4.4; [96]), while BAM file sorting and processing were managed via samtools [97].

Initial processing of the raw data yielded 115,743 SNPs. Joint genotyping was executed using the snpGBS and samtools packages, alongside the bcftools pipeline. Specifically, raw sequence alignments were processed using the bcftools mpileup command to generate genotype likelihoods, followed by multi-allelic variant calling and stringent filtering based on quality scores and entry formats. The generated datasets were converted into the required formats within the R environment (version 4.0.0; [98]). SNP quality control and filtering were performed using PLINK 1.9 [99,100,101]. Genotypes were filtered based on a minor allele frequency threshold (MAF ≥ 0.03) and a minimum call rate of 90% per sample. Following these stringent quality control steps, a total of 79,176 high-confidence SNPs were retained for downstream analyses.

### 2.5. Principal Component Analysis

Principal component analysis (PCA; [102]) based on a variance-standardized genetic relationship matrix was performed using PLINK 1.9 software to evaluate population structure. The visualization of the PCA results was generated within the R environment using the ggplot2 package [103,104].

### 2.6. GWAS Procedure

To detect associations between SNPs and the weight parameters of the whole egg and its constituent components in the F_2_ resource population, a linear regression analysis was performed using PLINK 1.9. The linear regression model accounted for standard statistical assumptions, including the linearity of the relationship between genotype and phenotype, independence of observations (controlled for population stratification via Principal Component Analysis, PCA), homoscedasticity (constant error variance), and normality of residuals. To correct for population stratification, the first four principal components (PC1–PC4) were included in the regression model as covariates. Additionally, fixed effects such as the sire effect and hatch batch were incorporated as covariates to control for non-genetic confounding factors.

The statistical significance of the SNP effects and the identification of genome-wide significant regions were evaluated using a null-hypothesis significance test with a Bonferroni-corrected threshold of *p* < 6.32 × 10^−7^. For SNPs that were reliably significant at the established significance threshold, in order to assess their possible pleiotropic effect, their associations with the studied traits were additionally considered at the suggestive level of *p* < 1.06 × 10^−6^.

The resulting data were visualized using Manhattan and Quantile-Quantile (Q-Q; Supplementary Figure S1) plots generated via the qqman package (version 0.1.9; [105,106]).

Candidate genes located within the genomic regions flanking the identified significant SNPs were retrieved using the CJA 2.0 reference genome assembly [93]. Functional annotation and gene ontology (GO) term enrichment analyses for the prioritized candidate genes (PCGs) that directly overlapped the exact SNP physical coordinates were performed using the Ensembl BioMart tool and the Database for Annotation, Visualization, and Integrated Discovery (DAVID Knowledgebase, version 2021/v2023q4; [107,108]).

## 3. Results

### 3.1. Phenotypic Characteristics of EW Parameters and Population Stratification

The phenotypic characteristics of the F_2_ resource population regarding the weight parameters of whole eggs and their primary constituent components (yolk, albumen, and eggshell) are summarized in Table 1.

The EW values within the evaluated resource population ranged from 9.500 to 14.605 g, with an average value of 12.62 ± 0.99 g. The weight parameters of individual egg components—yolk, total albumen, thick albumen, and eggshell—exhibited higher phenotypic variability relative to EW. Specifically, the coefficients of variation (CV) for YW, AW, TAW, and ESW reached 10.4%, 8.8%, 15.4%, and 9.9%, respectively. AYR, serving as an internal egg quality index, varied between 1.59 and 2.69, with a corresponding CV of 10.4%.

Table 2 presents the results of the GLM analysis, indicating the statistical significance levels of the fixed factors (Fisher’s F-statistic) included in the model on the variability of phenotypic traits (Main effects ANOVA). For the parental family group, a significant effect was established on the variation in TAW (*F* = 6.55, *p* = 0.0116). The incubation hatch batch of the contemporary group demonstrated a statistical trend for ESW (*F* = 1.77, *p* = 0.0722) and reached high statistical significance for the variability of TAW (*F* = 3.15, *p* = 0.0012). The sire effect of the F_2_ resource population significantly influenced ESW (*F* = 6.00, *p* = 0.0155) and approached significance as a trend for AYR (*F* = 3.69, *p* = 0.0569). The coefficient of determination (*R*^2^) of the GLM model explained between 13.2% and 30.6% of the phenotypic trait variance. The highest model accuracy regarding the analyzed group of factors on quail egg production parameters was achieved for ESW (*R*^2^ = 29.9%) and TAW (*R*^2^ = 30.6%).

The estimates of heritability coefficients and variance components are presented in Table 3. As anticipated, the heritability estimates for egg production and quality traits in the F_2_ resource quail population derived from crossing contrasting breeds exhibited varying degrees of variability. A low heritability estimate was obtained for EW (*h*^2^ = 0.077), indicating maximum phenotypic segregation and a disruption of linkage disequilibrium due to the interbreed cross between the parental lines of egg-meat Japanese and heavy meat-type Texas White quails. Conversely, ESW, YW, and AYR displayed substantially higher heritability values of 0.488, 0.237, and 0.323, respectively, suggesting a more stable genetic architecture for these traits. Interestingly, despite the pronounced differences in body weight between the founder breeds, ESW demonstrated the highest and most conservative heritability pattern, which likely indicates that this trait is less influenced by a highly polygenic architecture.

In contrast, a moderate heritability coefficient was observed for YW, whereas the heritability estimates for total AW and TAW were low, at 0.021 and 0.082, respectively. These differences could be attributed to the distinct physiological processes underlying the synthesis of protein and lipid components in the avian body. Interestingly, the heritability estimate for AYR was higher than those of its individual constituent parameters (*h*^2^ = 0.323). This higher heritability highlights its potential utility in breeding selection strategies (e.g., selection toward an egg-type profile by decreasing the ratio, or toward a meat-type profile by increasing it) and enables a more precise evaluation of individual birds within the resource population.

Analysis of phenotypic correlations among egg production traits in the resource quail population revealed the expected high positive relationships between EW and the weight parameters of individual egg components (Table 4). The highest phenotypic correlation was observed between EW and AW (*r* = 0.927), indicating the predominant contribution of albumen mass to total EW. The correlation coefficients of EW with other egg components, namely YW, TAW, and ESW, were lower, at 0.829, 0.496, and 0.696, respectively. Among the weight parameters of the individual egg components themselves—albumen, thick albumen, yolk, and eggshell—the correlation coefficients varied moderately, ranging from 0.320 to 0.575.

The genetic relationships among EW and its constituent egg components in the resource quail population exhibited varying degrees of correlation (Supplementary Table S1). The genetic relationships between EW—acting as the primary selection trait—and ESW or YW exhibited the highest positive values, at 0.932 and 0.953, respectively. However, the genetic correlations of EW with TAW, AW, and the AYR index were either strongly negative (ranging from −0.870 to −0.658) or entirely absent (*r_g_* = 0.031). This pattern aligns with the classical inverse genetic relationship observed between YW and AW parameters (with *r_g_* values ranging from −0.800 to −0.266). Additionally, an extremely close genetic correlation was observed between ESW and YW (*r_g_* = 0.982). Concurrently, negative genetic relationships were established between ESW and AW (ranging from −0.848 to −0.312) as well as the AYR index (*r_g_* = −0.968). Notably, a high negative correlation was also detected between YW and the AYR index (*r_g_* = −0.978). Finally, a strong positive genetic correlation was found between TAW and AW (*r_g_* = 0.604), while their respective correlations with the AYR index were 0.864 and 0.459, indicating that positive selection for these specific traits could effectively increase the albumen proportion in quail eggs.

**Figure 1:**
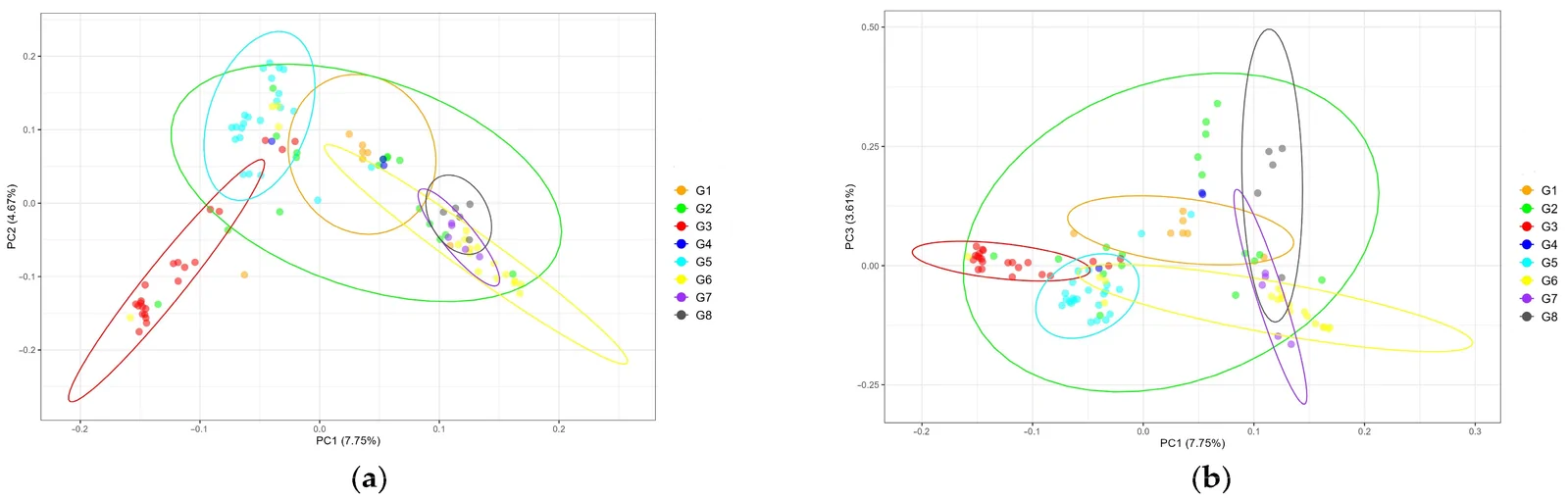
Principal component analysis (PCA) plots representing the genetic structure of the F_2_ Japanese quail resource population. (**a**) PCA plot in the plane of the first (PC1) and second (PC2) principal components; *X*-axis: PC1, *Y*-axis: PC2. (**b**) PCA plot in the plane of the first (PC1) and third (PC3) principal components; *X*-axis: PC1, *Y*-axis: PC3. G1–G8, groups of F_2_ females based on their respective F_1_ paternal lineages. Individuals belonging to different sire-derived groups are designated by distinct colors.

Assessment of the genetic structure of the evaluated F_2_ resource population via PCA revealed the distribution of individuals into several overlapping clusters. Depending on the specific F_1_ sires utilized to generate the resource population, multiple overlapping groups were observed in the PC1–PC2 and PC1–PC3 projections, as illustrated in Figure 1a,b.

### 3.2. GWAS Analysis

**Figure 2:**
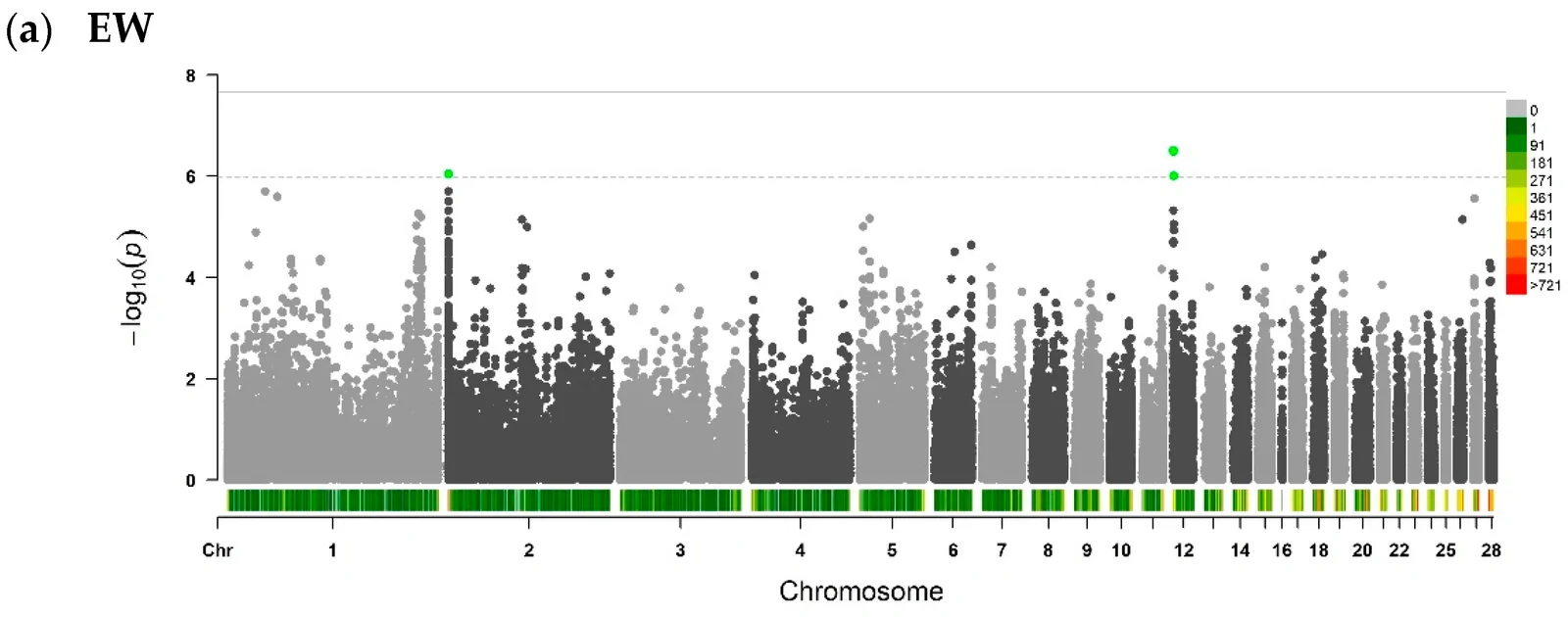

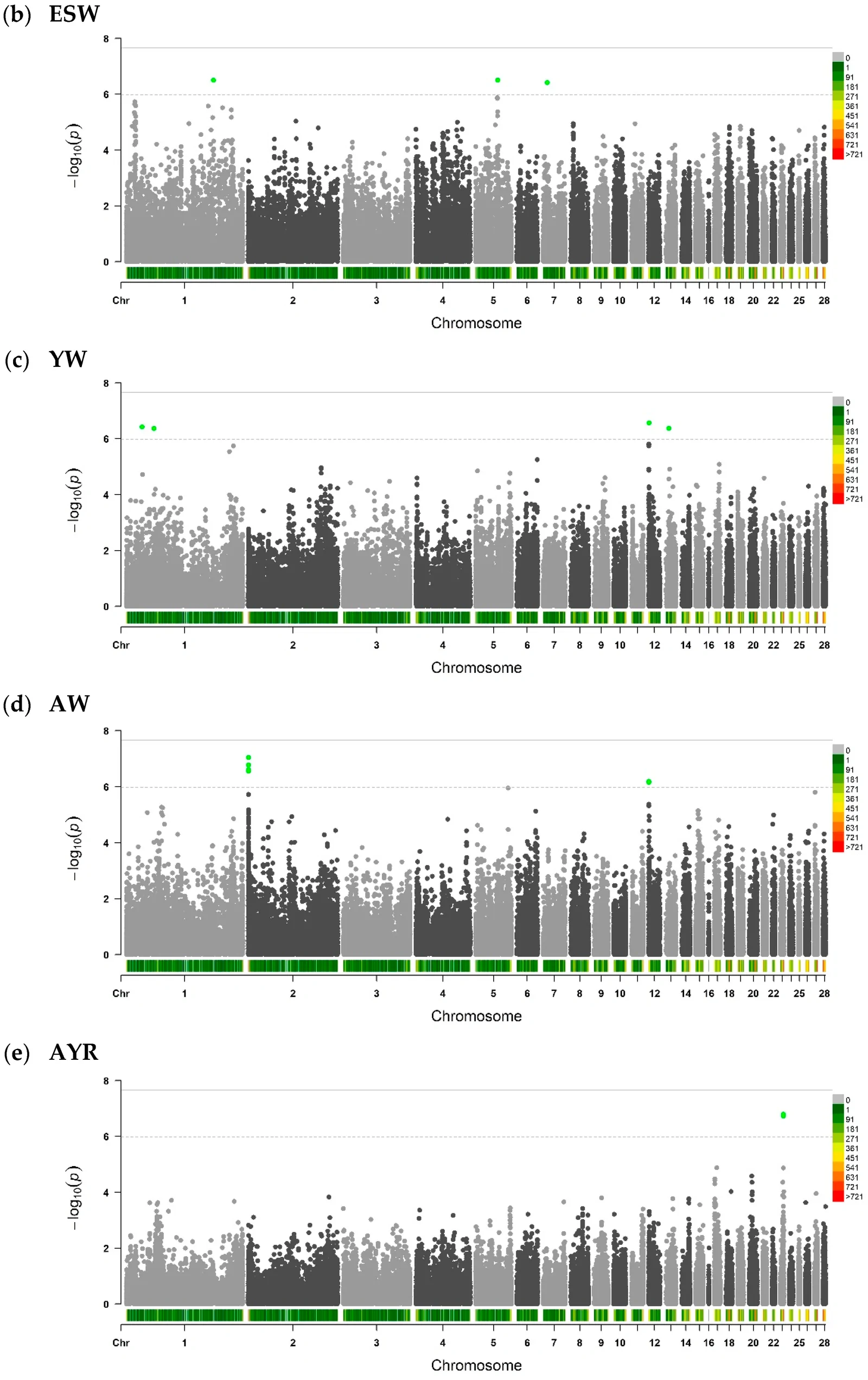
Manhattan plots displaying the genome-wide association study results for the evaluated egg weight and quality traits in the F_2_ quail resource population: (**a**) Egg weight (EW), (**b**) eggshell weight (ESW), (**c**) yolk weight (YW), (**d**) albumen weight (AW), and (**e**) albumen-to-yolk ratio (AYR). The Manhattan plots illustrate the genome-wide distribution of single nucleotide polymorphisms (SNPs) across the Japanese quail chromosomes (1–28) plotted against their statistical significance (−log_10_(*p*)). The dashed horizontal line represents the suggestive significance threshold (*p* < 1.06 × 10^−6^), and the solid horizontal line denotes the genome-wide significance threshold (*p* < 1.26 × 10^−8^). Significantly associated single nucleotide polymorphisms exceeding the threshold of *p* < 1.06 × 10^−6^ are highlighted in green. The color scale for the *X*-axis shows the density of detected SNPs (0 to >721) on individual chromosomes.

The GWAS results for EW, as well as the weight metrics and ratio of the primary egg components (i.e., AYR) in the evaluated F_2_ resource population, are presented in Figure 2 and Supplementary Figure S1.

Based on the GWAS results for EW and the weight metrics and ratio of egg’s constituent components, a total of 16 significantly associated SNPs were identified at the established Bonferroni-corrected genome-wide significance threshold of *p* < 6.32 × 10^−7^. For three of these 16 markers (12:309207, 12:335220, and 12:470023), additional significant associations with the investigated traits were established at the suggestive significance level of *p* < 1.06 × 10^−6^.

These variants were mapped across 7 of the 28 evaluated chromosomes: CJA1 (4 SNPs), CJA2 (4 SNPs), CJA5 (1 SNP), CJA7 (1 SNP), CJA12 (3 SNPs), CJA13 (1 SNP), and CJA23 (2 SNPs). The chromosomal distribution of these SNPs relative to each evaluated trait is summarized in Table 5.

The highest number of significant SNPs was detected for YW and AW (five and six SNPs, respectively), while the lowest number was observed for AYR with two SNPs (Table 2). Notably, several pleiotropic SNPs displayed significant associations with multiple evaluated traits. Specifically, three SNPs on chromosome CJA12 were identified as pleiotropic loci shared across distinct trait combinations: EW and AW (12:309207 and 12:335220) and EW and YW (12:470023). For the TAW trait, no genome-wide significant SNP associations were detected at the pre-established significance threshold used in this study.

### 3.3. Candidate Gene Identification

Within the genomic windows flanking the identified significant SNPs (SNP position ± 0.2 Mb), a total of 88 candidate genes were identified (Table 6).

Notably, seven genes directly overlapped the exact physical coordinates of these SNPs. They were designated as PCGs associated with the weight parameters of the whole egg and its primary components in laying quails: *NTN4* (netrin 4), *CLDN10* (claudin 10), *AGAP3* (ArfGAP with GTPase domain, ankyrin repeat and PH domain 3), *DUSP19* (dual specificity phosphatase 19), *STAB1* (stabilin 1), *SFMBT1* (Scm-like with four MBT domains 1), and *RNF130* (ring finger protein 130) (Table 4). These PCGs were mapped across five chromosomes: CJA1 (2 genes), CJA2 (1 gene), CJA7 (1 gene), CJA12 (2 genes), and CJA13 (1 gene). Among the seven PCGs, the identified SNPs were located within intronic regions for four genes (*CLDN10*, *AGAP3*, *STAB1*, and *RNF130*) and were situated within coding sequences (CDS) for three candidate genes (*NTN4*, *DUSP19*, and *SFMBT1*). Two genes localized on chromosome CJA12 exhibited significant pleiotropic associations with a subset of the traits evaluated in this study; specifically, the *STAB1* gene was associated with both EW and AW, while the *SFMBT1* gene was linked to YW and EW. Furthermore, two distinct SNPs associated with AW were found to reside within the genomic region of the *AGAP3* gene (Table 7).

GO enrichment analysis of the identified PCGs revealed that these loci are involved in the proteasome-mediated ubiquitin-dependent protein catabolic process, protein dephosphorylation, regulation of MAP kinase activity, cell migration, cell adhesion, regulation of DNA-templated transcription, GTP binding, protein binding, histone binding, calcium ion binding, and hyaluronic acid binding (Supplementary Table S2).

### 3.4. Allelic Variants of SNPs and Candidate Genes Underlying Phenotypic Variation in the Evaluated Traits

**Figure 3:**
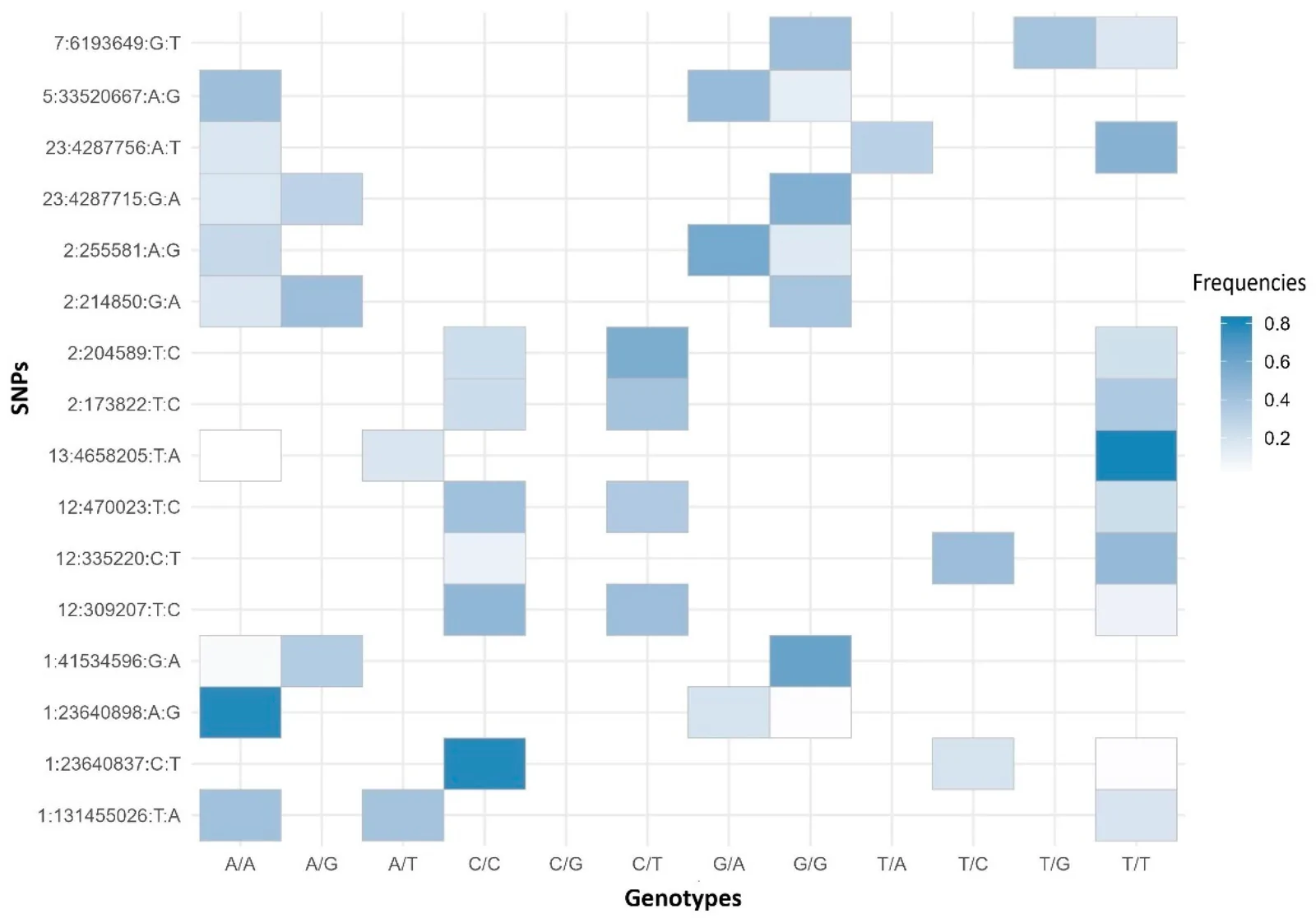
Genotype frequency heatmap for significant single nucleotide polymorphisms (SNPs) associated with egg weight and quality traits in the F_2_ Japanese quail resource population. *X*-axis: allelic combinations per SNP locus; *Y*-axis: 16 SNPs.

**Figure 4:**
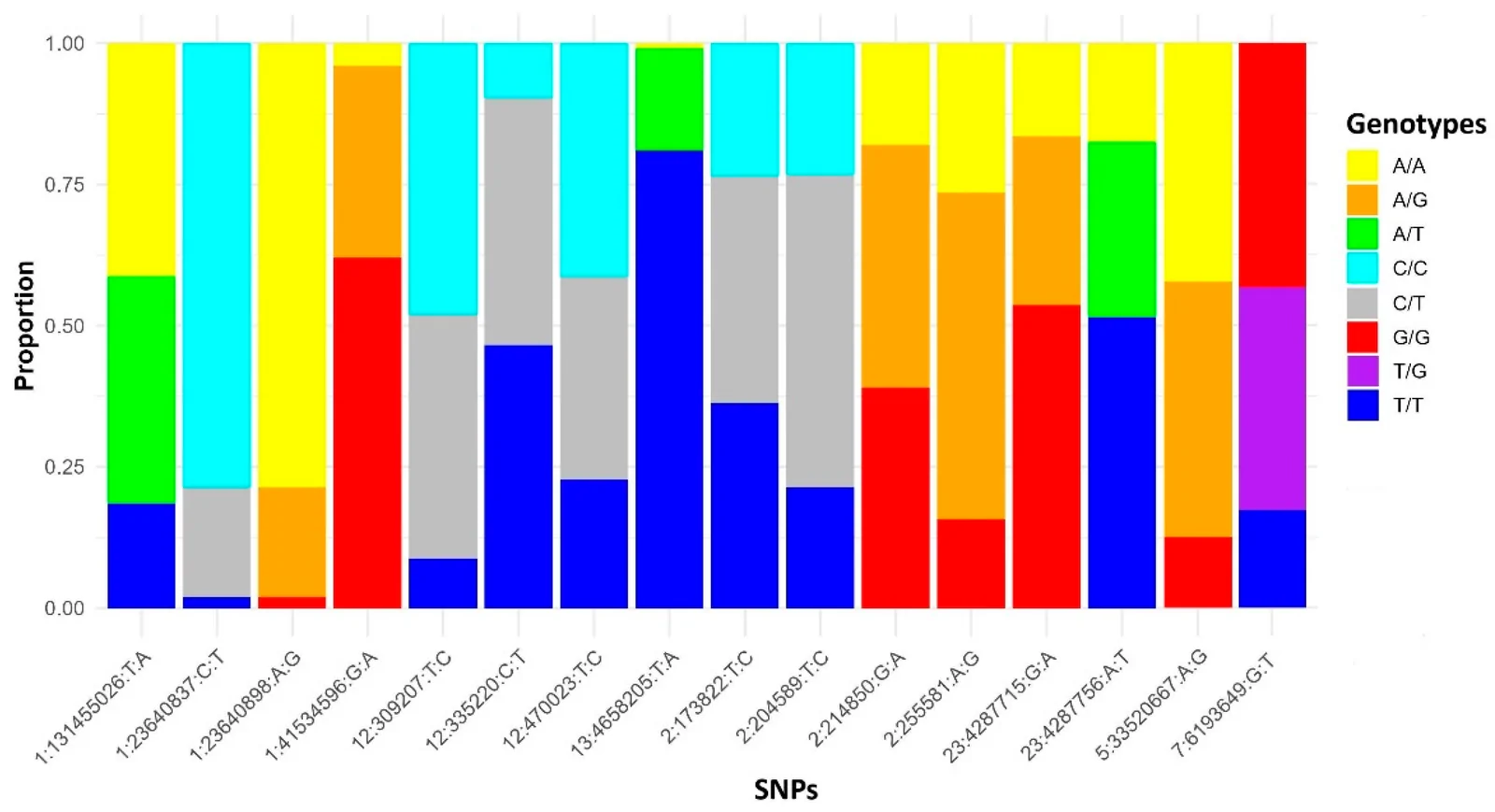
Genotype composition per significant single nucleotide polymorphism (SNP). It shows a proportionate distribution of genotypes for SNPs associated with egg weight and quality traits in the evaluated F_2_ Japanese quail resource population. *X*-axis: 16 SNPs; *Y*-axis: percentage of genotypes at individual SNP loci.

The genotype frequencies for the significant SNPs associated with the weight parameters of whole eggs and their components in the evaluated F_2_ resource population are illustrated in Figure 3 and Figure 4. For all 16 significant SNPs identified in this study, the F_2_ individuals exhibited three distinct allelic combinations (genotypes).

**Figure 5:**
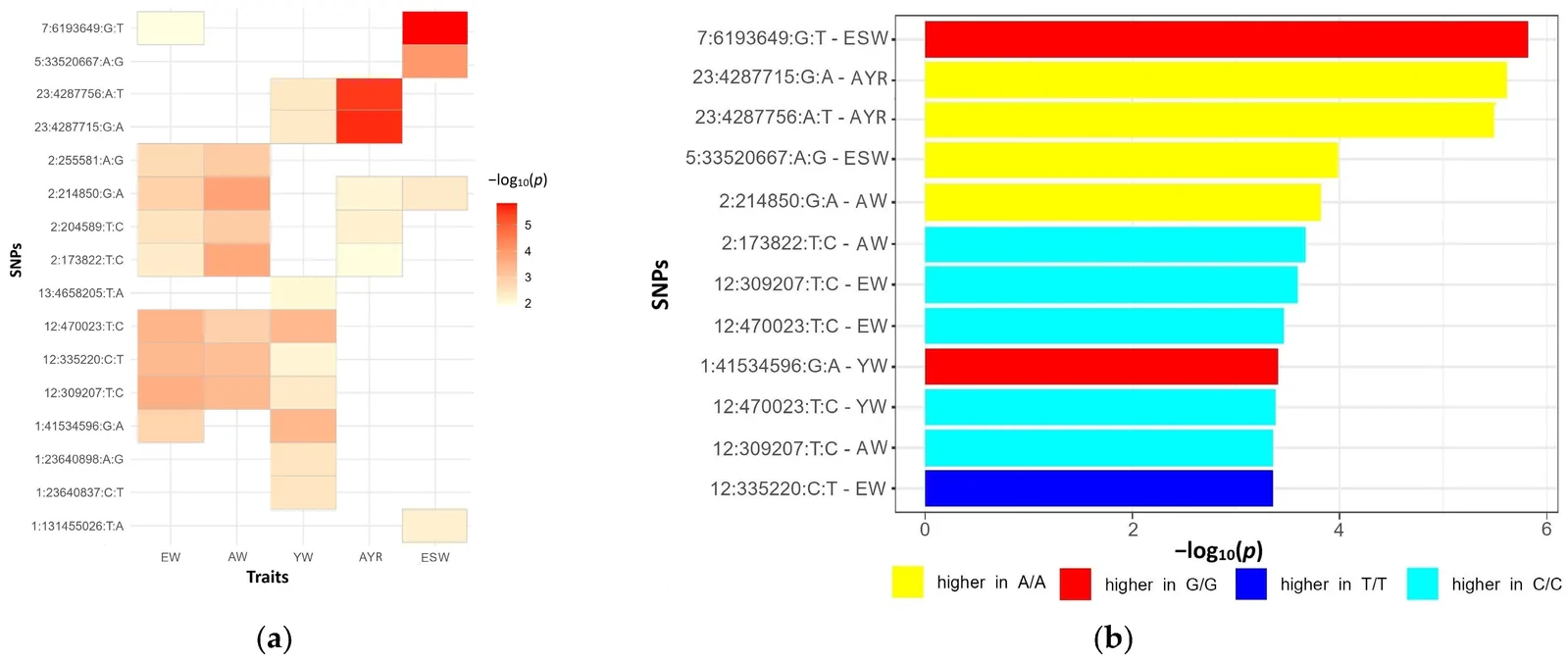
Statistical significance of single nucleotide polymorphism (SNP) associations with egg weight and quality traits in the F_2_ Japanese quail resource population. (**a**) Significance profiles of SNP associations for the evaluated traits at false discovery rate < 0.05; *X*-axis: significantly associated SNPs, *Y*-axis: evaluated phenotypic traits. (**b**) Ranking of the identified top 12 significant SNP associations according to their statistical significance level; *X*-axis: specific SNP–trait associations, *Y*-axis: significance level (−log_10_(*p*)); distinct colors designate the different genotypes associated with elevated phenotypic values for the evaluated traits. EW, egg weight; ESW, eggshell weight; AW, albumen weight; YW, yolk weight; AYR, albumen-to-yolk ratio.

Figure 5 summarizes the statistical significance of the SNP associations with the evaluated EW, egg component weights, and component ratio in the F_2_ resource population, alongside the specific allelic variants at these SNP positions that underlie high phenotypic values for the evaluated traits. The most statistically robust associations with EW metrics were detected for SNPs mapped to chromosomes CJA7 (7:6193649, linked to ESW) and CJA23 (23:4287715 and 23:4287756, linked to AYR).

**Figure 6:**
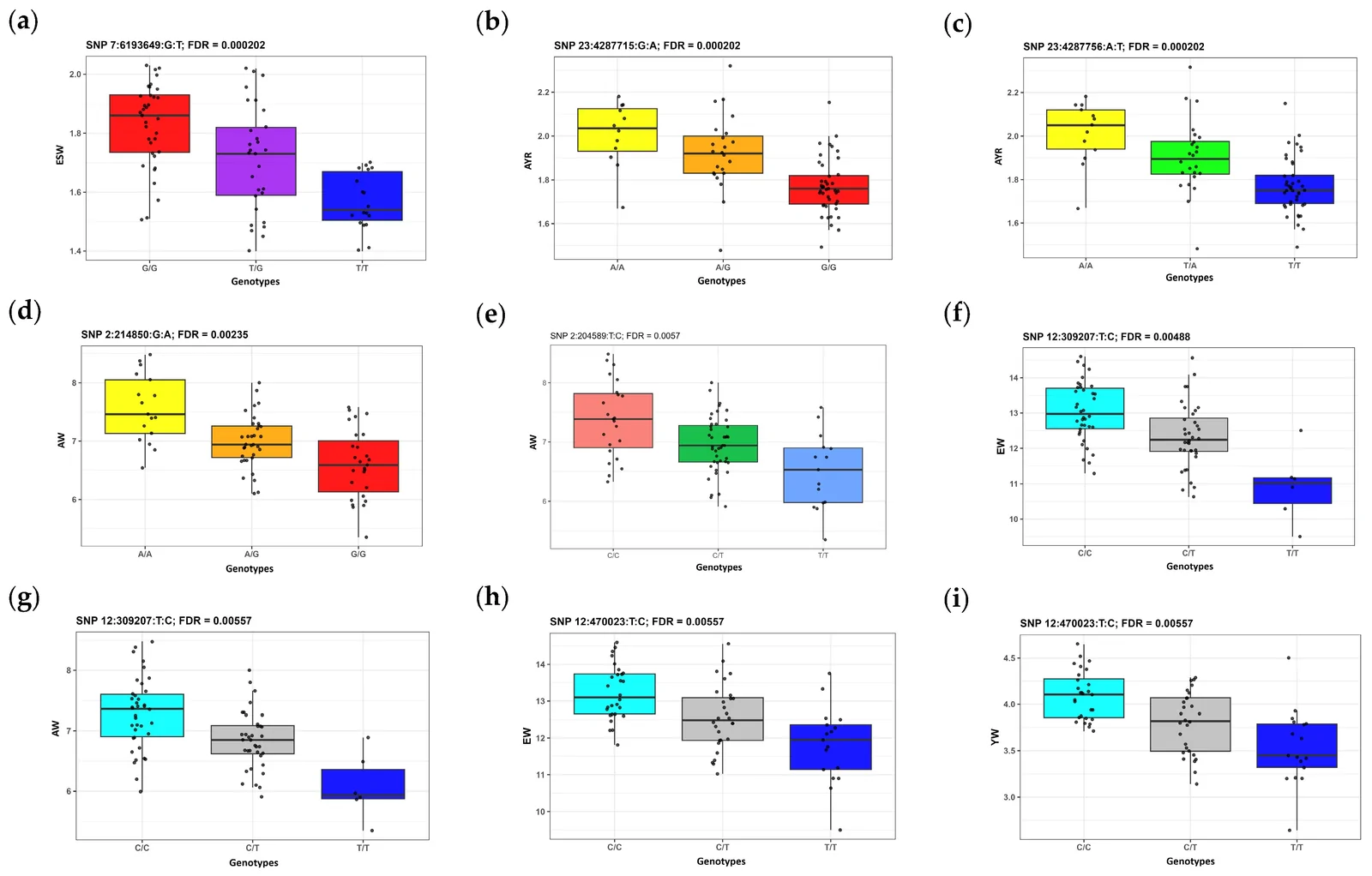
Whole egg weight, component weights, and component ratio in the F_2_ Japanese quail resource population stratified by genotype: (**a**) ESW by the *DUSP19* gene (7:6193649); (**b**) AYR by SNP 23:4287715; (**c**) AYR by SNP 23:4287756; (**d**) AW by the *AGAP3* gene (2:204589); (**e**) AW by the *AGAP3* gene (2:214850); (**f**) EW by the *STAB1* gene (12:309207); (**g**) AW by the *STAB1* gene (12:309207); (**h**) EW by the *SFMBT1* gene (12:470023); and (**i**) YW by the *SFMBT1* gene (12:470023). EW, egg weight; AW, albumen weight; YW, yolk weight; ESW, eggshell weight; AYR, albumen-to-yolk ratio; FDR, false discovery rate.

The SNP at position 7:6193649 is located within CDS of the *DUSP19* gene and is significantly associated with ESW. Within the evaluated quail population, the frequencies of the GG, GT, and TT genotypes at this locus were 0.43, 0.35, and 0.22, respectively. Elevated ESW significantly correlated with the GG genotype (Figure 6a, Supplementary Table S3). The SNPs at positions 23:4287715 and 23:4287756 are localized within intergenic regions and displayed significant associations with AYR. High phenotypic values for this index were observed in quails carrying the AA genotype at these loci (Figure 6b,c).

Additionally, attention should be drawn to gene-associated SNPs that demonstrated pleiotropic effects across distinct trait groups, as well as multiple significant SNPs residing within the boundaries of a single gene. Four such SNPs were identified in this study: two SNPs localized within the *AGAP3* gene (2:204589 and 2:214850), and two pleiotropic SNPs located within the *STAB1* (12:309207) and *SFMBT1* (12:470023) genes.

At the two *AGAP3* loci (2:204589 and 2:214850), the respective frequencies for the CC/CT/TT and AA/AG/GG genotypes in the F_2_ resource population were 0.28/0.53/0.19 and 0.22/0.44/0.34. The CC genotype at locus 2:204589 and the AA genotype at locus 2:214850 significantly correlated with increased AW (Figure 6d,e, Supplementary Table S3).

The CC, CT, and TT genotype frequencies for the two pleiotropic loci within *STAB1* (12:309207) and *SFMBT1* (12:470023) were 0.47/0.45/0.08 and 0.38/0.38/0.24, respectively. Compared to the TT and CT alternatives, the CC genotype at these loci dictated significantly higher phenotypic values for EW, as well as AW (for locus 12:309207) and YW (for locus 12:470023) (Figure 6f–i, Supplementary Table S3).

## 4. Discussion

In the current phase of poultry breeding, advancing the genetic potential of existing and emerging commercial lines and crosses [109,110,111,112,113] relies increasingly on integrating molecular and genomic tools into breeding programs. These technologies facilitate the prediction of laying performance, enabling targeted selection to establish highly productive flocks [114,115,116,117]. The Japanese quail serves as an exceptional model species for such genomic investigations, building upon a historical foundation of research into genetically controlled phenotypic and biochemical traits [118,119,120]. In this study, we utilized GBS data to evaluate the genomic associations of EW and its primary components (AW, ESW, TAW, YW, and AYR) in a custom-designed F_2_ resource population. Crossbred F_2_ resource populations represent powerful and informative livestock models for molecular mapping studies aimed at discovering genetic markers for economically valuable traits. They are widely utilized across various livestock species [121,122,123,124], including poultry [125,126], employing GWAS strategies for genetic mapping [127,128,129,130]. The fundamental strategy of establishing a resource population by crossing divergent breeds with distinct extremes of a target trait maximizes phenotypic variance within a relatively small, manageable population. Here, the resource population was generated by crossing two quail breeds with contrasting egg production characteristics, including EW: the Japanese breed and the Texas White breed (with average EW being 11 and 13 g, respectively) [76]. The F_2_ resource quail population generated from these contrasting founder breeds and investigated in this study was characterized by relatively high variability in both EW (ranging from 9.5 to 14.6 g) and the weight metrics of the primary egg components, specifically YW (2.6–4.6 g), TAW (2.8–6.2 g), AW (5.8–9.6 g), and ESW (0.98–1.42 g) (Table 1). The subpopulation structure of the resulting resource quail population was robustly confirmed via PCA (Figure 1) and subsequently accounted for as a covariate framework during the GWAS analysis.

In the current study, we identified 16 genome-wide significant SNPs and 88 candidate genes, including 7 PCGs located directly at the SNP physical positions, associated with EW, component weights ESW, YW and AW, and the AYR index. Notably, a substantial proportion of the identified significant SNPs were mapped within intergenic and intronic sequences (66%, 12 SNPs). Although these non-coding genomic regions do not alter protein structures, an increasing body of literature highlights their critical role in modulating gene expression and transcriptional regulation [131,132]. Among the 12 SNPs located in intergenic and intronic sequences, two variants (12:309207 and 12:335220) exhibited pleiotropic effects, showing robust significant associations with a subset of traits, namely EW and AW, which aligns with the high phenotypic correlation established between EW and AW in this study (*r* = 0.927). Specifically, SNP 12:309207 was localized within an intronic region of the *STAB1* gene on chromosome CJA12. Furthermore, four significant SNPs were identified within the introns of the *AGAP3* (2:204589 and 2:214850, associated with AW), *CLDN10* (1:131455026, associated with ESW), and *RNF130* (13:4658205, associated with YW) genes. GO enrichment analysis indicated that the candidate loci *STAB1*, *AGAP3*, *CLDN10*, and *RNF130* are functionally involved in the proteasome-mediated ubiquitin-dependent protein catabolic process, GTP binding, GTPase activator activity, GTPase activity, protein binding, cell adhesion, calcium ion binding, and hyaluronic acid binding (Supplementary Table S3).

A comprehensive review of publicly available databases and literature revealed no prior reports confirming a direct functional impact of the *STAB1*, *AGAP3*, *CLDN10*, and *RNF130* genes on EW parameters or internal egg composition in quails. Nevertheless, several independent studies have documented significant associations between SNPs residing within or flanking these specific loci and egg formation dynamics, alongside other economically important traits across various livestock species, including poultry.

Firstly, the *CLDN10* gene, a member of large claudin family [133], warrants specific attention. It encodes claudin-10, an essential protein involved in the passive paracellular transport of calcium ions (Ca^2+^) across the uterine epithelium in laying hens [134,135]. Calcium absorbed in the small intestine and transported to the uterus is critically essential for eggshell formation and biomineralization, processes that require intensive calcium carbonate deposition during the ovulatory cycle. Sun et al. [136] investigated the role of *CLDN10* in eggshell pigmentation abnormalities (mottling) in brown-egg-laying hens. Based on single-cell RNA sequencing of individual cells isolated from the uterus and in situ RNA hybridization on uterine histological sections, the expression of *CLDN10* within the epithelial lining of the eggshell gland was robustly confirmed. Eggshell mottling is a color defect characterized by altered pigmentation and compromised eggshell ultrastructure, typically manifesting as localized thickening at the site of the defect. Given that the uterus/eggshell gland directly modulates key external quality attributes—including shell thickness and mechanical strength—these functional insights provide strong biological validation for our observed association between the *CLDN10* locus and ESW in the F_2_ quail population, since eggshell thickness and breaking strength directly correlate with total shell weight [43]. Furthermore, Luo et al. [137] identified a significant association between *CLDN10* and both feed efficiency and feed conversion ratio in Wenchang chickens (broilers) during the 89–113 day growth cycle. Nutritional regimens and feed utilization efficiency are key deterministic factors governing the realization of productive potential in livestock species. In poultry breeding, enhanced feed conversion efficiency underlies high-performance profiles in both meat and egg lineages, directly dictating EW and the individual weights of internal egg components.

Beyond *CLDN10*, the *STAB1* gene has been functionally linked to immune responsiveness in chickens [138], as well as meat quality attributes in swine (meat tenderness [139]; backfat thickness [140]) and poultry (meat color [141]). Additionally, statistically robust associations have been documented for the *AGAP3* gene relative to reproductive traits in water buffaloes [142] and swine [143], and environmental adaptation profiles in goats [144]. Host immunity directly modulates overall avian health, whereas effective environmental adaptation mechanisms indicate superior stress resilience. Both physiological health status and stress resilience represent critical baseline drivers governing quantitative performance metrics in layers, including egg production rate, EW, and internal egg quality traits. Finally, the physiological roles of the *STAB1* and *RNF130* genes have been verified in lipoprotein metabolism [145], specifically regarding the regulatory clearance of hepatic low-density lipoprotein receptors and plasma low-density lipoprotein cholesterol concentrations [146,147,148]. The functional expression of *RNF130* has also been demonstrated in the chicken liver [149]. Because lipoproteins serve as the core biochemical building blocks constituting avian egg yolk [30], these metabolic pathways provide a logical genetic mechanism explaining the association of these candidate loci with internal egg composition.

Among the 16 genome-wide significant SNPs identified herein, only three were localized within CDS of candidate genes, specifically *NTN4* (1:41534596, linked to YW), *DUSP19* (7:6193649, linked to ESW), and *SFMBT1* (12:491438, linked to EW and YW). Functional annotation indicated that these polymorphic loci are biologically involved in protein dephosphorylation, regulation of MAP kinase activity, basement membrane assembly, cell migration, substrate adhesion-dependent cell spreading, protein binding, and the regulation of DNA-templated transcription (Supplementary Table S3).

The *DUSP19* gene encodes dual-specificity phosphatase 19, an enzyme belonging to the dual-specificity phosphatase/mitogen-activated protein kinase phosphatase (DUSP/MKP) subfamily. It participates in modulating mitogen-activated protein kinase (MAPK) cascades by suppressing c-Jun N-terminal kinase (JNK) and p38 signaling pathways, particularly during cellular stress responses. Previous functional studies have documented the involvement of *DUSP19* in the pathogenesis of osteoarthritis, where a downregulation of *DUSP19* accelerates chondrocyte apoptosis via JNK pathway activation [150]. Furthermore, *DUSP19* expression levels negatively correlate with leptin concentrations; conversely, the overexpression of *DUSP19* induced by elevated leptin levels protects chondrocytes against apoptosis via JNK dephosphorylation. Crucially, extensive research has established that the leptin system heavily influences growth traits, reproductive efficiency, and egg production profiles across diverse poultry species, including quails [151,152,153,154]. This biological link suggests a potential indirect or upstream regulatory effect of *DUSP19* on EW and individual egg component weights. Additionally, a non-synonymous mutation within the coding sequence of *DUSP19* could compromise its intrinsic phosphatase activity, triggering aberrant hyperactivation of JNK/p38 and worsening cellular stress. Persistent hyperactivation of JNK within ovarian or oviductal cells may induce local apoptotic cascades, disrupt normal follicular maturation, and ultimately suppress egg-laying intensity in poultry.

The *NTN4* gene encodes netrin-4, a member of the netrin protein family that shares structural homology with laminins [155]. Netrin-4 stimulates neurite outgrowth from the olfactory bulb, modulates neuronal migration, regulates angiogenesis, and serves as a fundamental structural component of the basement membranes in vasculature and various parenchymal organs [155,156]. The physiological expression of *NTN4* has been robustly demonstrated in the kidneys, heart, aorta, mammary glands, and ovaries [155], pointing toward a critical homeostatic role in avian folliculogenesis and ovarian vascularization. Consequently, a mutation within the coding sequence of *NTN4* could alter the growth kinetics and vascularization of preovulatory follicles, thereby directly influencing the egg production rate and quality metrics of laying quails.

The *SFMBT1* gene regulates transcription in both somatic and germ cells by forming a stable corepressor complex with LSD1 and CoREST. Upon binding to target promoters, it recruits associated proteins to induce chromatin compaction and transcriptional repression [157]; it is also involved in modulating myogenic pathways [158]. The protein encoded by *SFMBT1* contains an MBT domain and shares homology with the *Drosophila* Scm-like with four MBT domains (SFMBT) protein, a Polycomb group member driving the epigenetic regulation of gene expression [157]. A potential non-synonymous substitution within the MBT domain of *SFMBT1* could disrupt the recognition of methylated histones and destabilize the Polycomb repressive complex, leading to the aberrant derepression of downstream target genes regulating cellular differentiation. Within the avian ovary, these altered epigenetic processes could trigger disruptions in folliculogenesis, thereby compromising the normal oviposition cycle in quails. Furthermore, independent mapping studies have documented significant associations between SNPs within the *SFMBT1* gene and behavioral phenotypes, such as aggressive behavior in meat-type broiler roosters [159], as well as meat quality traits, including breast meat color in chickens [141], and body conformation metrics in beef cattle [160].

The findings of this study significantly expand fundamental insights into the polygenic mechanisms governing the expression and realization of egg production potential in laying quails [161,162,163]. The candidate loci identified herein, which directly overlap genome-wide significant SNPs associated with EW and egg quality components, may exert both direct and indirect functional impacts on these quantitative traits. To validate the biological contributions of these PCGs fully to quail egg production, further large-scale replication studies integrating advanced GWAS approaches, whole-genome resequencing, and multi-omics frameworks are warranted [164,165,166].

## 5. Conclusions

In this study, a GWAS analysis was performed using GBS data to investigate the genetic architecture of EW and its primary constituent components—albumen, yolk, and eggshell—in an F_2_ Japanese quail resource population. Our examination identified 16 genome-wide significant SNPs and 88 candidate genes, including seven PCGs overlapping the exact physical coordinates of these SNPs. These genomic loci exhibited robust statistical associations with EW (3 SNPs; *STAB1* and *SFMBT1* genes), AW (7 SNPs; *AGAP3* and *STAB1* genes), TAW (2 SNPs), ESW (3 SNPs; *CLDN10* and *DUSP19* genes), YW (5 SNPs; *NTN4*, *SFMBT1*, and *RNF130* genes), and AYR (2 SNPs). Notably, three pleiotropic variants on chromosome CJA12 were associated with distinct trait combinations (12:309207 and 12:335220 with EW and AW; 12:470023 with EW and YW), while the *AGAP3* gene harbored two specific SNPs (2:204589 and 2:214850) significantly linked to AW. We mapped the population-specific allele frequencies at these polymorphic loci and identified the specific genotypes underlying high phenotypic values for the evaluated traits. Taken together, these experimental data significantly contribute to clarifying the polygenic mechanisms governing egg composition and productive potential in laying quails. The identified SNPs and PCGs provide an essential baseline for future population-specific validation studies to explore their potential utility as prospective selection markers in quail breeding programs aimed at optimizing egg quality metrics.

## Figures and Tables

**Table 1:** Descriptive statistics ^1^ of egg weight and quality traits in the F_2_ quail resource population (*n* = 102).

Trait	Mean	SD	Min	Max	CV, %
Egg weight, g	12.624	0.987	9.500	14.605	7.8
Eggshell weight, g	1.213	0.121	0.977	1.424	9.9
Yolk weight, g	3.837	0.400	2.640	4.646	10.4
Thick albumen weight, g	4.635	0.715	2.791	6.183	15.4
Albumen weight, g	7.574	0.663	5.803	9.583	8.8
Albumen-to-yolk ratio	1.99	0.21	1.59	2.69	10.4

^1^ SD, standard deviation; min, minimum; max, maximum; CV, coefficient of variation.

**Table 2:** General Linear Model (GLM) analysis of fixed effects influencing phenotypic egg production and quality traits in the F_2_ resource quail population.

Traits	Effects						*R* ^2^
	Parent Group Effect		Hatch Batch Effect		Sire Effect		
	*F*	*p*-Value	*F*	*p*-Value	*F*	*p*-Value	
Egg weight	0.67	0.4129	1.06	0.3940	0.59	0.4451	0.142
Eggshell weight	1.70	0.1950	1.77	0.0722 ^t^	6.00	0.0155 *	0.299 ***
Yolk weight	0.05	0.8243	1.23	0.2798	2.53	0.1138	0.182
Thick albumen weight	6.55	0.0116 *	3.15	0.0012 **	0.71	0.4004	0.306 ***
Albumen weight	1.46	0.2283	0.96	0.4782	0.04	0.8372	0.132
Albumen-to-yolk ratio	1.54	0.2163	1.53	0.1348	3.69	0.0569 ^t^	0.175

Note: ^t^ statistical trend (*p* < 0.10); * significant at *p* < 0.05; ** highly significant at *p* < 0.01; *** very highly significant at *p* < 0.01; *R*^2^, coefficient of determination of the GLM model for each respective trait.

**Table 3:** Variance components and heritability estimates (*h*^2^) for egg production and quality traits in the F_2_ resource quail population.

Traits	Genetic Variance (*VarA*)	Residual Variance (*VarE*)	Heritability (*h*^2^)
Egg weight	0.0944	1.1320	0.077
Eggshell weight	0.0084	0.0088	0.488
Yolk weight	0.0464	0.1496	0.237
Thick albumen weight	0.0328	0.3668	0.082
Albumen weight	0.0103	0.4665	0.021
Albumen-to-yolk ratio	0.0145	0.0303	0.323

Note: *VarA*, additive genetic variance; *VarE*, residual variance; *h*^2^, narrow-sense heritability coefficient.

**Table 4:** Phenotypic correlations among egg weight and quality components in the F_2_ resource quail population.

Traits	EW	ESW	YW	TAW	AW	AYR
**EW**	1.000					
**ESW**	0.696	1.000				
**YW**	0.829	0.544	1.000			
**TAW**	0.496	0.320	0.353	1.000		
**AW**	0.927	0.564	0.575	0.500	1.000	
**AYR**	−0.139	−0.116	−0.660	0.046	0.225	1.000

Note: EW, egg weight; ESW, eggshell weight; YW, yolk weight; TAW, thick albumen weight; AW, total albumen weight; AYR, albumen-to-yolk ratio.

**Table 5:** Chromosomal distribution of significant single nucleotide polymorphisms (SNPs; *p* < 1.06 × 10^−6^) associated with egg weight and quality traits in the F_2_ Japanese quail resource population.

Trait	Number of SNPs	Chromosomes (No. of SNPs)
Egg weight	3	CJA 12 (3 SNPs)
Eggshell weight	3	CJA1 (1 SNP), CJA5 (1 SNP), CJA7 (1 SNP)
Yolk weight	5	CJA1 (3 SNPs), CJA12 (1 SNP), CJA13 (1 SNP)
Albumen weight	6	CJA2 (4 SNPs), CJA12 (2 SNPs)
Albumen-to-yolk ratio	2	CJA23 (2 SNPs)

**Table 6:** List of significant single nucleotide polymorphisms (SNPs; *p* < 1.06 × 10^−6^) and candidate genes associated with egg weight and quality traits in the F_2_ Japanese quail resource population.

Trait ^1^	SNP (Chromosome: Position)	Region	*p*-Value	Candidate Genes
EW	12:309207	Intron	3.07 × 10^−7^	*RPL29*, *TNNC1*, *NISCH*, *STAB1*, *NT5DC2*, *PBRM1*, *GNL3*, *GLT8D1*, *SPCS1*, *NEK4*, *ITIH3*, *MUSTN1*, *SFMBT1*
	12:335220	Intergenic	3.26 × 10^−7^	*RPL29*, *TNNC1*, *NISCH*, *STAB1*, *NT5DC2*, *PBRM1*, *GNL3*, *GLT8D1*, *SPCS1*, *NEK4*, *ITIH3*, *MUSTN1*, *SFMBT1*
	12:470023	CDS ^2^	9.78 × 10^−7^	*STAB1*, *NT5DC2*, *PBRM1*, *GNL3*, *GLT8D1*, *SPCS1*, *NEK4*, *ITIH3*, *MUSTN1*, *SFMBT1*, *PRKCD*, *HYAL1*, *HYAL2*, *TUSC2*, *ZMYND10*, *NPRL2*, *TMEM115*, *CACNA2D2*
ESW	1:131455026	Intron	3.15 × 10^−7^	*UGGT2*, *DNAJC3*, *DZIP1*, *CLDN10*, *ABCC4*
	5:33520667	Promoter	3.15 × 10^−7^	*SLC25A21*, *MIPOL1*, *FOXA1*
	7:6193649	CDS	3.83 × 10^−7^	*NUP35*, *DUSP19*, *NCKAP1*, *FRZB*, *MAP2*
YW	1:23640837	Intergenic	3.75 × 10^−7^	*FOXP2*, *PPP1R3A*
	1:23640898	Intergenic	3.75 × 10^−7^	*FOXP2*, *PPP1R3A*
	1:41534596	CDS	4.33 × 10^−7^	*FGD6*, *VEZT*, *METAP2*, *USP44*, *NTN4*, *SNRPF*, *AMDHD1*, *HAL*, *LTA4H*, *ELK3*, *CDK17*
	12:470023	CDS	2.70 × 10^−7^	*STAB1*, *NT5DC2*, *PBRM1*, *GNL3*, *GLT8D1*, *SPCS1*, *NEK4*, *ITIH3*, *MUSTN1*, *SFMBT1*, *PRKCD*, *HYAL1*, *HYAL2*, *TUSC2*, *ZMYND10*, *NPRL2*, *TMEM115*, *CACNA2D2*
	13:4658205	Intron	4.20 × 10^−7^	*RUFY1*, *HNRNPH1*, *CANX*, *MAML1*, *MGAT4B*, *SQSTM1*, *MRNIP*, *TBC1D9B*, *RNF130*, *FLT4*, *HMGXB3*, *CSF1R*, *PDGFRB*, *CDX1*, *SLC6A7*
AW	2:173822	Promoter	8.97 × 10^−8^	*CHPF2*, *ASB10*, *GBX1*, *AGAP3*, *TMUB1*, *FASTK*, *ASIC3*, *ABCB8*, *ATG9B*, *NOS3*, *KCNH2*, *AOC1*
	2:204589	Intron	2.44 × 10^−7^	*CHPF2*, *ASB10*, *GBX1*, *AGAP3*, *TMUB1*, *FASTK*, *ASIC3*, *ABCB8*, *ATG9B*, *NOS3*, *KCNH2*, *AOC1*, *RARRES2*
	2:214850	Intron	1.64 × 10^−7^	*CHPF2*, *ASB10*, *GBX1*, *AGAP3*, *TMUB1*, *FASTK*, *ASIC3*, *ABCB8*, *ATG9B*, *NOS3*, *KCNH2*, *AOC1*, *RARRES2*
	2:255581	Promoter	2.77 × 10^−7^	*CHPF2*, *ASB10*, *GBX1*, *AGAP3*, *TMUB1*, *FASTK*, *ASIC3*, *ABCB8*, *ATG9B*, *NOS3*, *KCNH2*, *AOC1*, *RARRES2*
	12:309207	Intron	6.67 × 10^−7^	*RPL29*, *TNNC1*, *NISCH*, *STAB1*, *NT5DC2*, *PBRM1*, *GNL3*, *GLT8D1*, *SPCS1*, *NEK4*, *ITIH3*, *MUSTN1*, *SFMBT1*
	12:335220	Intergenic	6.41 × 10^−7^	*RPL29*, *TNNC1*, *NISCH*, *STAB1*, *NT5DC2*, *PBRM1*, *GNL3*, *GLT8D1*, *SPCS1*, *NEK4*, *ITIH3*, *MUSTN1*, *SFMBT1*
AYR	23:4287715	Intergenic	1.62 × 10^−7^	*C23H1orf94*, *CSMD2*, *SMAP2*, *RIMS3*, *NFYC*, *KCNQ4*, *TINAGL1*, *PEF1*, *ADGRB2*, *PTP4A2*, *KHDRBS1*, *TMEM39B*
	23:4287756	Intergenic	1.84 × 10^−7^	*C23H1orf94*, *CSMD2*, *SMAP2*, *RIMS3*, *NFYC*, *KCNQ4*, *TINAGL1*, *PEF1*, *ADGRB2*, *PTP4A2*, *KHDRBS1*, *TMEM39B*

^1^ EW, egg weight; AW, albumen weight; YW, yolk weight; ESW, eggshell weight; AYR, albumen-to-yolk ratio. ^2^ CDS, coding sequences.

**Table 7:** List of prioritized candidate genes (PCGs; *p* < 1.06 × 10^−6^) associated with the weight of the whole egg and its primary components in the F_2_ Japanese quail resource population.

Chromosome	PCG	SNP Position	Allele	Region	*p*-Value	Trait ^1^
CJA1	*NTN4*	41,534,596	G/A	CDS ^2^	4.33 × 10^−7^	YW
	*CLDN10*	131,455,026	T/A	Intron	3.15 × 10^−7^	ESW
CJA2	*AGAP3*	204,589	T/C	Intron	2.44 × 10^−7^	AW
		214,850	G/A	Intron	1.64 × 10^−7^	AW
CJA7	*DUSP19*	6,193,649	G/T	CDS	3.83 × 10^−7^	ESW
CJA12	*STAB1*	309,207	T/C	Intron	3.07 × 10^−7^	EW, AW
	*SFMBT1*	470,023	T/C	CDS	2.70 × 10^−7^	EW, YW
CJA13	*RNF130*	4,658,205	T/A	Intron	4.20 × 10^−7^	YW

^1^ EW, egg weight; AW, albumen weight; YW, yolk weight; ESW, eggshell weight. ^2^ CDS, coding sequences.

## Data Availability

The original contributions presented in this study are included in the article/Supplementary Materials. Further inquiries can be directed to the corresponding authors. The sequence data are accessible to readers on request.

## Supplementary Materials

The following supporting information can be downloaded at: https://www.mdpi.com/article/10.3390/vetsci13090967/s1, Figure S1: Quantile–quantile (Q–Q) plots for the GWAS results in the F_2_ Japanese quail resource population. (a) EW, egg weight; (b) ESW, eggshell weight; (c) YW, yolk weight; (d) AW, albumen weight; (e) AYR, albumento- yolk ratio.; Table S1: Genetic correlations (*r_g_*) among EW and its constituent components in the F_2_ resource quail population; Table S2: Gene ontology (GO) term enrichment analysis at the positions of the determined SNPs in F_2_ quails of the resource population; Table S3: Egg weight components in F_2_ quails of the resource population depending on the single nucleotide polymorphism (SNP) genotype for the prioritized candidate genes (PCG) *DUSP19*, *AGAP3*, *STAB1* and *SFMBT1*.

## Abbreviations

The following abbreviations are used in this manuscript:

GWAS	Genome-wide association study
SNP	Single nucleotide polymorphism
EW	Egg weight
ESW	Eggshell weight
AW	Albumen weight
TAW	Thick albumen weight
YW	Yolk weight
AYR	Albumen-to-yolk ratio
CJA	*Coturnix japonica* chromosome
QTLs	Quantitative trait loci
GBS	Genotyping-by-sequencing
MAF	Minor allele frequency
PCA	Principal component analysis
Q-Q	Quantile-Quantile
GO	Gene ontology
PCG	Prioritized candidate gene
SD	Standard deviation
CV	Coefficient of variation
PC	Principal component
*NTN4*	Netrin 4
*CLDN10*	Claudin 10
*AGAP3*	ArfGAP with GTPase domain, ankyrin repeat and PH domain 3
*DUSP19*	Dual specificity phosphatase 19
*STAB1*	Stabilin 1
*SFMBT1*	Scm-like with four MBT domains 1
*RNF130*	Ring finger protein 130
JNK	c-Jun N-terminal kinase
