# Supplementary material for: Genome-Wide Association Studies Reveal Key Candidate Genes for Egg Weight and Quality Components in an F2 Japanese Quail Resource Population
Source: Vet Sci. 2026 Sep 15;13(9):967. doi: 10.3390/vetsci13090967 (PMC13611952; doi:10.3390/vetsci13090967)
Supplement: Supplementary file 1 [file vetsci-13-00967-s001.zip › Supplementary Figure S1.pdf]

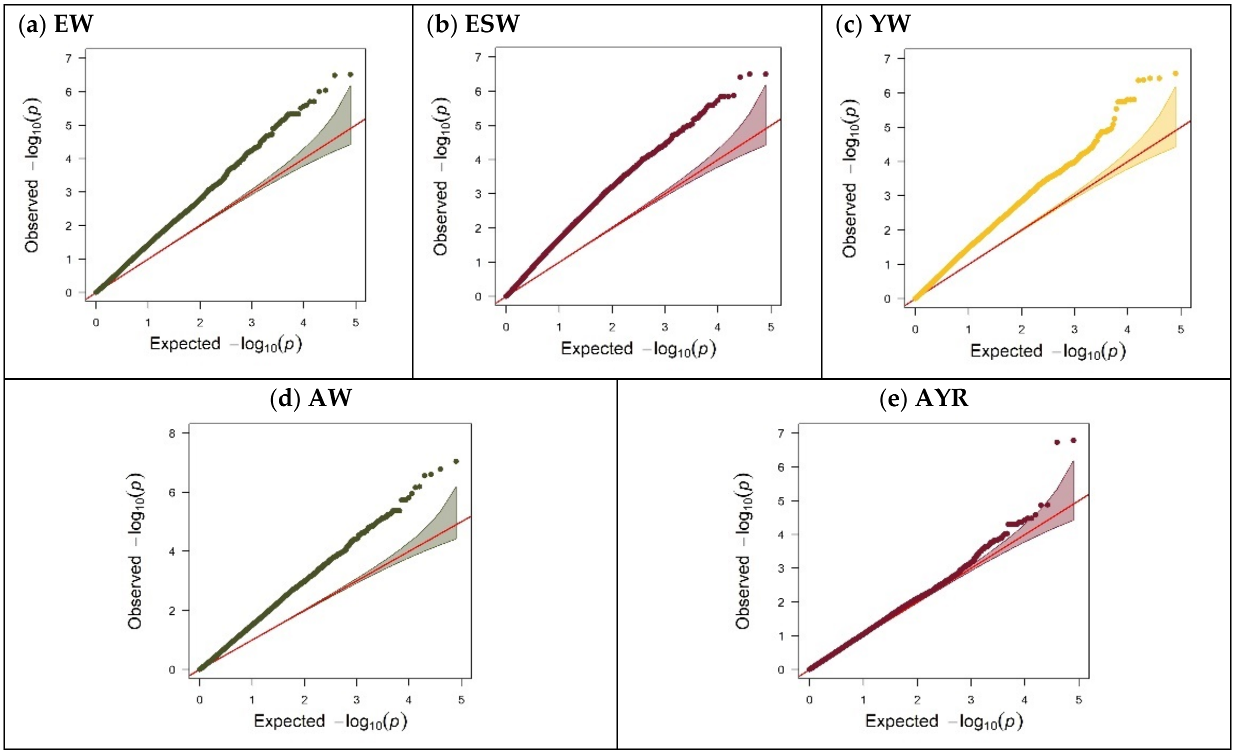

Supplementary Figure S1. Quantile–quantile (Q–Q) plots for the GWAS results in the F_2 Japanese quail resource population. (**a**) EW, egg weight; (**b**) ESW, eggshell weight; (**c**) YW, yolk weight; (**d**) AW, albumen weight; (**e**) AYR, albumen-to-yolk ratio.
