# Supplementary material for: Genome-Wide Association Studies Reveal Key Candidate Genes for Egg Weight and Quality Components in an F2 Japanese Quail Resource Population
Source: Vet Sci. 2026 Sep 15;13(9):967. doi: 10.3390/vetsci13090967 (PMC13611952; doi:10.3390/vetsci13090967)
Supplement: Supplementary file 1 [file vetsci-13-00967-s001.zip › Supplementary Table S1.pdf]

Supplementary Table S1. Genetic correlations (r_g) among EW and its constituent components in the F_2 resource quail population.

Traits	EW	ESW	YW	TAW	AW	AYR
EW	—					
ESW	0.932	—				
YW	0.953	0.982	—			
TAW	−0.658	−0.848	−0.800	—		
AW	0.031	−0.312	−0.266	0.604	—	
AYR	−0.870	−0.968	−0.978	0.864	0.459	—

Note: EW, egg weight; ESW, eggshell weight; YW, yolk weight; TAW, thick albumen weight; AW, total albumen weight; AYR, albumen-to-yolk ratio.
