# Supplementary material for: Genome-Wide Association Studies Reveal Key Candidate Genes for Egg Weight and Quality Components in an F2 Japanese Quail Resource Population
Source: Vet Sci. 2026 Sep 15;13(9):967. doi: 10.3390/vetsci13090967 (PMC13611952; doi:10.3390/vetsci13090967)
Supplement: Supplementary file 1 [file vetsci-13-00967-s001.zip › Supplementary Table S3.pdf]

Supplementary Table S3. Egg weight components in F_2 quails of the resource population depending on the single nucleotide polymorphism (SNP) genotype for the prioritized candidate genes (PCG) *DUSP19*, *AGAP3*, *STAB1* and *SFMBT1*.

Trait [1]	PCG	SNP (chromosome: position)	Genotype	*n*	Fre-quency	Mean ± SD [2]	Difference between genotypes		
							Genotypes	%	*p*-value
EW	*STAB1*	12:309207	CC	47	0.48	13.04 ± 0.83	CC − CT	5.8	0.001
			CT	45	0.45	12.33 ± 0.91	CT − TT	12.9	0.001
			TT	7	0.07	10.92 ± 1.00	TT − CC	19.4	0.001
	SFMBT1	12:470023	CC	35	0.38	13.23 ± 0.741	CC − CT	5.3	0.001
			CT	35	0.38	12.57 ± 0.897	CT − TT	6.4	0.005
			TT	21	0.24	11.81 ± 1.035	TT − CC	12.0	0.001
ESW	*DUSP19*	7:6193649	GG	44	0.43	1.280 ± 0.099	GG − TG	6.8	0.001
			TG	36	0.35	1.198 ± 0.087	TG − TT	9.2	0.001
			TT	22	0.22	1.097 ± 0.067	TT − GG	16.7	0.001
AW	*AGAP3*	2:204589	CC	29	0.28	7.974 ± 0.737	CC − CT	6.6	0.005
			CT	53	0.53	7.482 ± 0.521	CT − TT	6.7	0.005
			TT	19	0.19	7.011 ± 0.665	TT − CC	13.7	0.001
		2:214850	AA	21	0.21	8.167 ± 0.693	AA − AG	8.4	0.001
			AG	42	0.44	7.532 ± 0.484	AG − GG	6.2	0.001
			GG	34	0.35	7.094 ± 0.625	GG − AA	15.1	0.001
	STAB1	12:309207	CC	47	0.48	7.812 ± 0.605	CC − CT	5.4	0.005
			CT	45	0.45	7.412 ± 0.630	CT − TT	13.0	0.001
			TT	7	0.07	6.559 ± 0.554	TT − CC	19.1	0.001
YW	*SFMBT1*	12:470023	CC	35	0.38	4.095 ± 0.260	CC − CT	7.9	0.001
			CT	35	0.38	3.795 ± 0.332	CT − TT	7.1	0.01
			TT	21	0.24	3.543 ± 0.307	TT − CC	15.6	0.001

[1] EW, egg weight (g); AW, albumen weight (g); YW, yolk weight (g); ESW, eggshell weight (g).
